# Analysis of cardiac manifestation and treatment of multisystem inflammatory syndrome in children related to SARS-CoV-2

**DOI:** 10.17305/bjbms.2022.7820

**Published:** 2023-03-16

**Authors:** Stasa Krasic, Sanja Ninic, Sergej Prijic, Sasa Popovic, Srdjan Pasic, Gordana Petrovic, Boris Zec, Snezana Ristic, Dejan Nesic, Luka Nikolic, Vladislav Vukomanovic

**Affiliations:** 1 Cardiology Department, Faculty of Medicine, Mother and Child Health Institute of Serbia, University of Belgrade, Belgrade, Serbia; 2 Immunology Department, Faculty of Medicine, Mother and Child Health Institute of Serbia, University of Belgrade, Belgrade, Serbia; 3 Pediatric Clinic, University Clinical Centre of the Republic of Srpska, Banja Luka, Bosnia and Herzegovina; 4 Intensive Care Unit, Mother and Child Health Institute of Serbia, Belgrade, Serbia; 5 Institute of Medical Physiology, Faculty of Medicine, University of Belgrade, Belgrade, Serbia

**Keywords:** Acute myocardial dysfunction, shock, pediatric, severe acute respiratory syndrome coronavirus-2 (SARS-CoV-2), multisystem inflammatory syndrome in children (MIS-C)

## Abstract

Cardiovascular (CV) manifestations are common (35%–100%) in the multisystem inflammatory syndrome in children. Our study aimed to analyze treatment impact and CV involvement in patients with multisystem inflammatory syndrome in children. The retrospective cohort included 81 patients treated between April 2020 and December 2021 (9.3 ± 4.6 years). Elevated cardiac troponin I and pro-B-type natriuretic peptide were observed in 34.2% and 88.5% of patients, respectively. Myocardial dysfunction was observed in 50.6%. Children older than 10 years had a 4-fold increased risk of myocardial dysfunction (odds ratio [OR] 3.6, 95% confidence interval [CI] 1.4–8.9; *p* ═ 0.006). A moderate negative correlation was proved between left ventricular ejection fraction and C-reactive protein (rr ═ −0.48; *p* < 0.001). More than one-fifth of the patients presented with shock. Coronary artery dilatation was observed in 6.2% of patients. Mild pericardial effusion was detected in 27.1% of children. On standard electrocardiogram, 52.6% of children had negative T waves in the inferior and/or precordial leads; transient QTc prolongation was registered in 43% of patients. Treatment failure was observed in 19 patients. Patients initially treated with intravenous immunoglobulins had 10-fold higher chances for treatment failure than patients treated with corticosteroids (OR 10.6, 95% CI 3.18–35.35; *p* < 0.001). CV manifestations were observed in more than half of the patients, with acute myocardial dysfunction being the most common, especially in children older than 10 years. We established a negative association between the degree of elevation of inflammatory markers and left ventricular ejection fraction. Patients treated with intravenous immunoglobulins who had CV manifestations had treatment failures more frequently than patients treated with corticosteroids.

## Introduction

A multisystem inflammatory syndrome in children (MIS-C) temporarily associated with coronavirus disease 2019 (COVID-19) is a hyperinflammatory state caused by cytokine and chemokine storm following symptomatic or asymptomatic severe acute respiratory syndrome coronavirus-2 (SARS-CoV-2) infection in genetically suitable children. Viral-induced autoimmune reaction and tolerance deterioration cause cytokine and chemokine storm [1–3]. The current estimated incidence of MIS-C is 2%–6% in those younger than 21 years [4, 5]. Patients with MIS-C can present with various signs, symptoms, and severities; gastrointestinal (GI) and cardiovascular (CV) symptoms are the most common manifestations. Other common clinical presentations are non-exudative conjunctivitis (45%–56%), mucocutaneous rash (~60%), and neurologic symptoms (30%–58%) [5].

CV involvement appears in up to 67%–97% of children with MIS-C [4–8]. The cardiac manifestations include ventricular dysfunction, pericardial effusion, shock, coronary artery (CA) dilatations and aneurysms, conduction abnormalities, arrhythmias, and valvulitis. Literature reports that 50%–80% of patients present with either vasodilatory shock, cardiogenic shock, or a combination of both [4–9].

Based on treatment guidelines for Kawasaki disease (KD), multiple patients with MIS-C were treated with intravenous immunoglobulin (IVIG) alone or combined with corticosteroids (CS). Some authors suggest that adding CS to IVIG is associated with a shorter duration of fever and shorter recovery time of cardiac function in patients with MIS-C [8, 9].

Our study aimed to investigate the CV involvement in patients with MIS-C treated in our hospital. Additionally, the impact of initial therapy (IVIG or CS) on the disease outcome was analyzed.

## Materials and methods

### Study design

A retrospective study included children under 18 years of age with MIS-C associated with COVID-19 treated at the Mother and Child Health Institute from April 2020 to December 2021. The United States Centers for Disease Control and Prevention criteria were used to diagnose the MIS-C associated with COVID-19 [5]. In a mild to moderate form of the disease, patients did not require admission to the intensive care unit (ICU), did not require oxygen and ventilatory support, and did not require inotropic medications. Severe form implied severe organ injury, shock, and ICU admission. Clinical examination, laboratory analysis, and echocardiographic examination were performed in all patients on admission. A serological examination for SARS-CoV-2 was performed using the enzyme-immuno-essay (ELISA) and immunochromatography. We analyzed following CV manifestations and parameters of MIS-C: myocardial dysfunction, pericardial effusion, MIS-C shock, CA aneurysms, electrocardiographic (ECG) changes, as well as elevated cardio-specific markers (cardiac troponin I (cTnI) and pro-B-type natriuretic peptide [proBNP]).

Myocardial dysfunction included ventricular systolic function abnormalities (ejection fraction (EF) < 55%), edema of the interventricular septum and posterior wall, and elevated BNP levels. The presence of MIS-C shock was defined by the persistence of following criteria: (1) systolic arterial hypotension, (2) a drop in basal systolic blood pressure of at least 20%, or (3) the appearance of signs of peripheral hypoperfusion [9, 10].

Hepatitis was defined as an elevation of alanine aminotransferase (ALT) >40 IU/L and aspartate aminotransferase (AST) > 50 IU/L [24]. Gamma-glutamyl transferase (γGT) and bilirubin levels were measured in all patients with elevated ALT and AST.

The local Ethics Committee approved the study, which waived the requirement for informed parental consent (Approval No: 9/13).

### Treatment protocol

**Figure 1.**
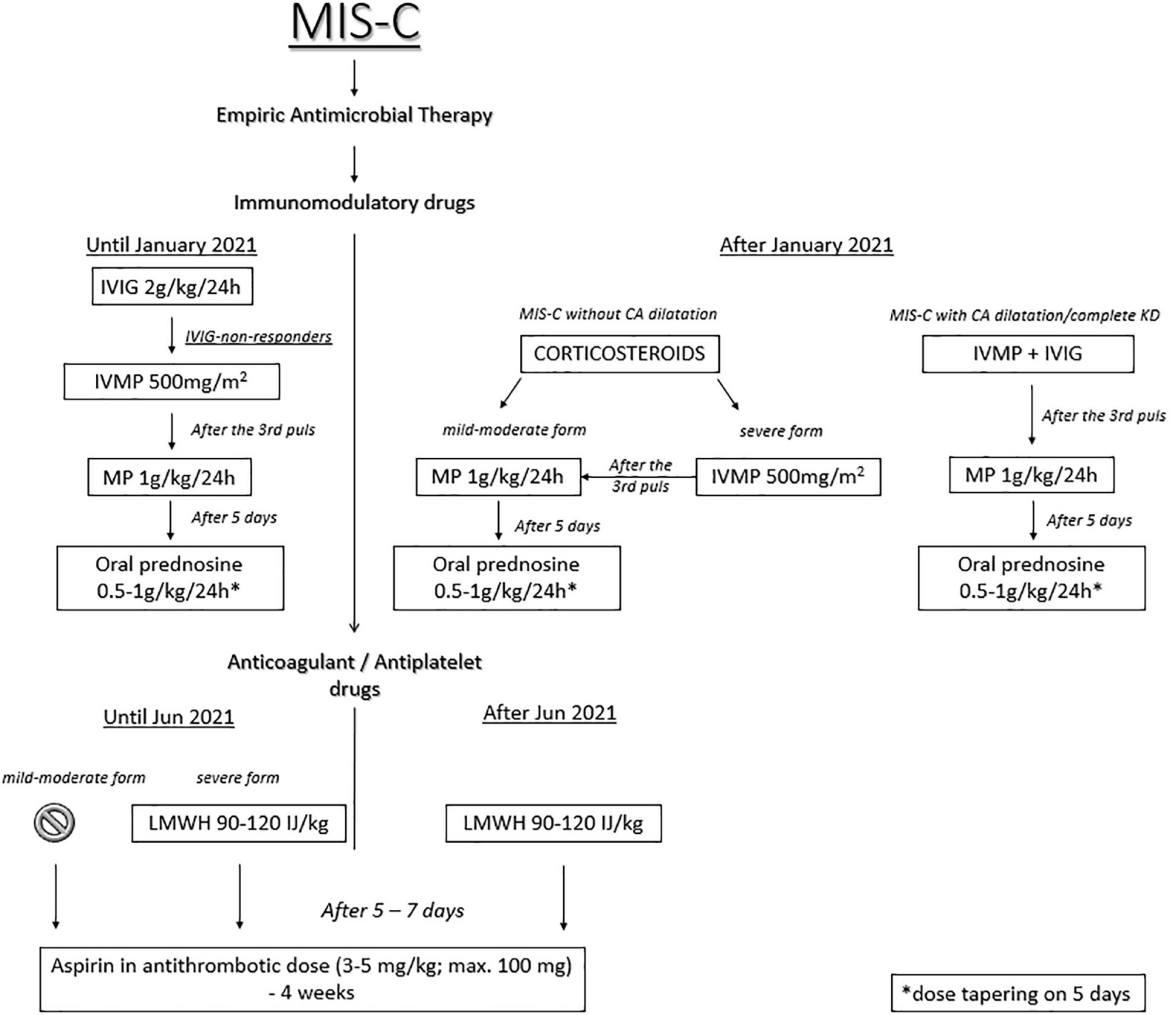
**The treatment protocol of our patients with MIS-C temporarily associated with COVID-19.** MIS-C: Multisystem inflammatory syndrome in children; IVIG: Intravenous immunoglobulins; IVMP: Intravenous methylprednisolone pulses; MP: Methylprednisolone; LMWH: Low-molecular-weight heparin; CA: Coronary artery.

Patients were treated with supportive and immunomodulatory drugs and received anticoagulant and antithrombotic therapy. Supportive treatment included intravenous hydration, diuretics, and angiotensin-converting enzyme (ACE) inhibitors. Patients with myocardial dysfunction and shock were treated with inotropes: dopamine, dobutamine, and milrinone [9, 10]. All patients admitted to the hospital received parenteral empiric antibiotic therapy after taking samples for blood and urine culture. Immunomodulatory treatment started shortly after admission. IVIGs (2 g/kg during 24 h) were the first used immunomodulatory drugs according to the KD treatment guidelines, and based on the same protocols. IVIG-non-responsive patients were treated with CS—three intravenous methylprednisolone (IVMP) pulses (500 mg/m^2^). After eight months of the MIS-C treatment experience (from January 2021), we observed that almost all patients were IVIG-non-responsive and required CS. As a result, we started treating all patients only with CS, while the combination of CS and IVIG was administered only to patients with CA dilatation. Patients with mild disease were treated with standard CS doses (1 mg/kg), while patients with the severe form received IVMP (500 mg/m^2^). After three IVMPs, patients received intravenous CS in standard doses (1 mg/kg) for up to seven days. After administering CS in standard doses, all patients were treated with oral CS (prednisone) with a gradual dose reduction. The treatment protocol is presented in Figure 1. Since we have changed the hospital treatment protocol, we wanted to compare whether there were differences in treatment failure depending on the therapy used. Treatment failure was defined as the fever occurrence (>38 ^∘^C) after 48 h of immunomodulatory therapy initiation or the development of MIS-C shock and persistence of acute left ventricular (LV) systolic dysfunction (EF <55%) [10].

Initially, only patients treated in the pediatric ICU (PICU) received prophylactic anticoagulant therapy (low-molecular-weight heparin [LMWH]), but because thrombosis occurred in a patient with a mild clinical presentation, we used LMWH in all patients with MIS-C. LMWH was used in the prophylactic dose (90–120 IU/kg) during 5–7 in-hospital days, and after this period, it was replaced with aspirin in antithrombotic doses (3–5 mg/kg; maximum dose 100 mg daily).

### Statistical analysis

The descriptive statistics included the mean, median, standard deviation (SD), the interquartile range (IQR), and total number and percentage (%) of the parameters monitored. The distribution difference of specific parameters among the groups tested was determined using the χ2 or Fisher’s test. Using the Shapiro–Wilk and Kolmogorov–Smirnov tests, we tested the normality of the distribution of the numerical variables. The groups were compared using the Student’s t-test and Mann–Whitney test. Paired t-tests and Wilcoxon tests were used to compare two related samples. Pearson or Spearman tests tested the correlation between vital parameters in different groups. We used binominal logistic regression analysis to explain the relationship between the dependent binary variable and the independent variables. The data were processed using statistical software SPSS 25.0 for Windows 10. All statistical methods were considered statistically significant if *p* ≤ 0.05.

## Results

The study included 81 patients, 47 males (58%) and 34 females (42%). The mean age was 9.3 (SD 4.6) years. Female patients were younger than males (median 8.2; IQR 4.8–11.1 years vs 10.3 (IQR 7.0–14.9) years; *p* ═ 0.04). Serological blood tests were positive in 77 patients (95%), while 6 (7.4%) had positive PCR or antigen tests from a nasopharyngeal swab on SARS-CoV-2.

**Figure 2.**
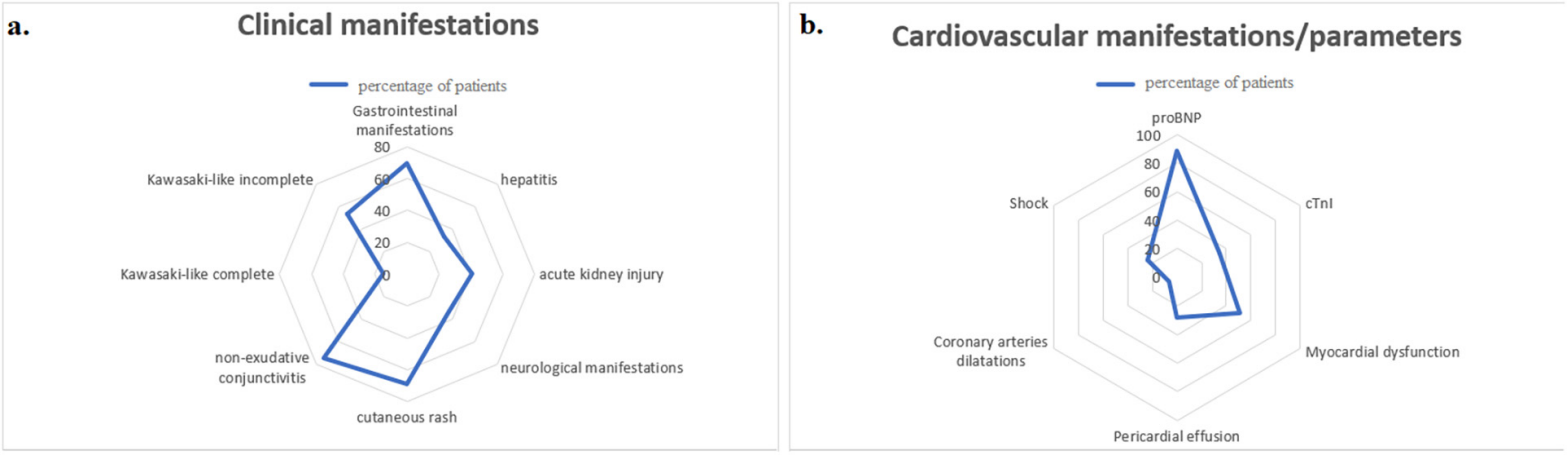
**Clinical, cardiovascular manifestations, and parameters of patients.** Radar graphs showing percentages of clinical (A) and cardiovascular manifestations (B). proBNP: pro-B-type natriuretic peptide; cTnI: Cardiac troponin I.

**Figure 3.**
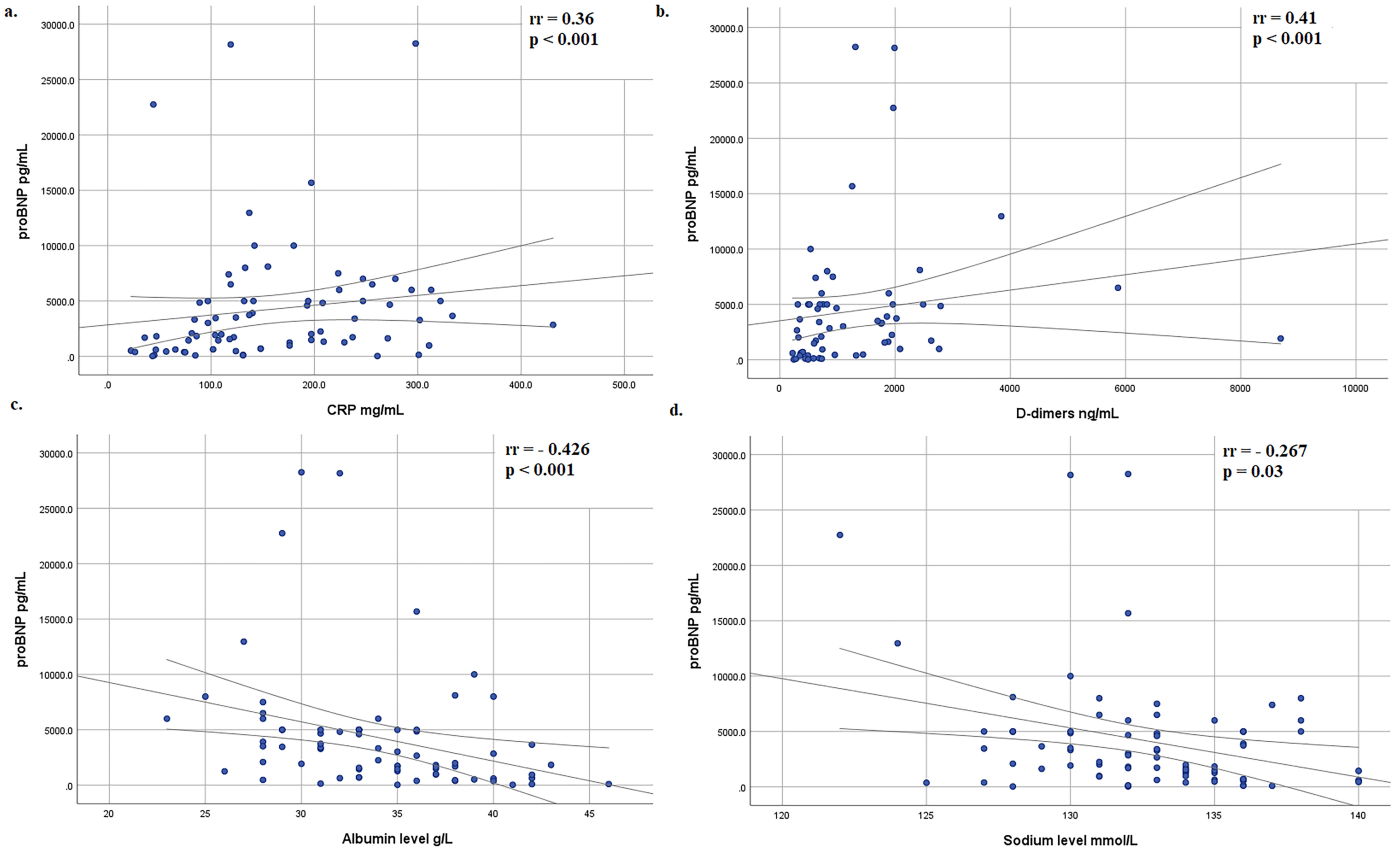
**Correlation between laboratory analysis at the admission.** A mild to moderate positive correlation was proven between: (A) proBNP level and CRP, (B) proBNP and D-dimer. A mild to moderate negative correlation was proven between: (C) proBNP and albumin; (D) proBNP and sodium level. proBNP: pro-B-type natriuretic peptide; CRP: C-reactive protein.

All patients had fever that lasted a median of 5 (IQR 4–6) days. Kawasaki-like disease had 55/81 patients (67.9%); complete Kawasaki-like disease had 12/55 patients (21.8%), while nonexudative conjunctivitis had 60/81 patients (74.1%). Most children had GI manifestations (56/81, 69.1%), following the kidney (32/81, 39.2%) and liver injury (27/81, 33.3%). Neurological manifestation had 29/81 patients (35.8%), out of which 2/29 (6.8%) had a stroke (Figure 2A). Three patients underwent appendectomies. Initial laboratory findings showed elevated proinflammatory markers (C-reactive protein [CRP], fibrinogen, D-dimer, and ferritin) and low serum sodium, phosphate, albumin, and platelet count (Table 1). Elevated cTnI and proBNP were observed in 34.2% and 88.5% of patients, respectively. Average proBNP was 4523.2 (SD 5374) pg/mL. In patients with elevated cTnI, median cTnI value was 0.59 (IQR 0.14–1.55) ng/mL. A mild to moderate positive correlation was proven between CRP and proBNP (rr ═ 0.36, *p* < 0.001). proBNP correlated positive with leukocyte count (rr ═ 0.297, *p* < 0.001) and D-dimer (rr ═ 0.41, *p* < 0.001), while proBNP negative correlated with albumin (rr ═ −0.426, *p* < 0.001) and sodium level (rr ═ −0.267, *p* ═ 0.03) (Figure 3). Level of the cTnI level on admission correlated positively with proBNP (rr ═ 0.27, *p* ═ 0.03). Patients with hepatitis had a higher level of serum γGT in comparison with patients without liver damage (median 48, IQR 23.5–90.0 vs median 19, IQR 12–36 IU/L; *p* ═ 0.007). Ursodeoxycholic acid was added in treatment of five patients, and only in patients with hepatitis (5/26 vs 0/55; *p* ═ 0.003).

Patients with GI manifestations had more frequently severe form of the disease and more frequently required ICU admission than patients without GI manifestations.

CV manifestations had 53/81 patients (64.6%) (Figure 2B). In those patients, higher serum concentration of CRP and D-dimer was observed in comparison with patients without CV manifestations (CRP 180.0, IQR 116–266.0, vs 96.9, IQR 69.7–123.0 mg/L; *p* < 0.001; D-dimer 1177, IQR 687.25–1985.75 vs 465, IQR 352–1574 ng/mL; *p* ═ 0.035).

**Figure 4.**
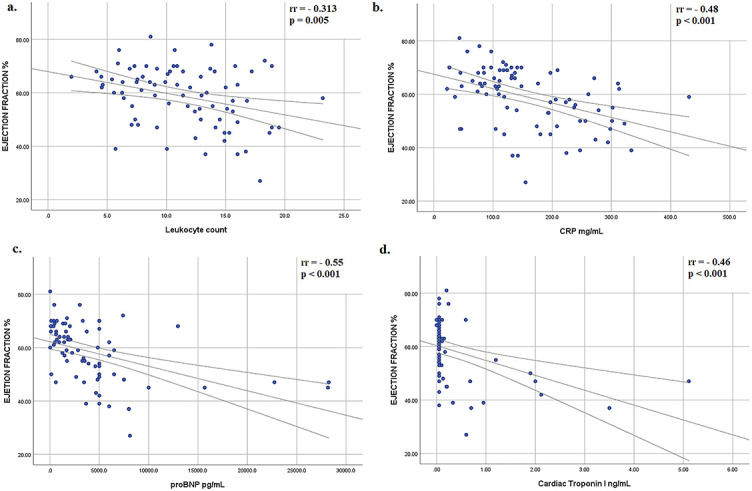
**Correlation between left ventricular ejection fraction and laboratory analysis at the admission**. A moderate negative correlation was proven between: (A) ejection fraction and leukocytes count; (B) ejection fraction and CRP; (C) ejection fraction and proBNP; (D) ejection fraction and cardiac troponin I. proBNP: pro-B-type natriuretic peptide; CRP: C-reactive protein.

Myocardial dysfunction was observed in half of all patients (50.6%), with an average LV EF of 50.5% (SD 8.7%). A limited number of patients had LV EF lower than 40% (6/81 patients). Children older than 10 years had an almost 4 times-fold increased chance for myocardial dysfunction (odds ratio [OR] 3.6, 95% CI 1.4–8.9; *p* ═ 0.006). There was no difference between genders in the frequency of myocardial dysfunction. Patients with myocardial dysfunction had more frequently GI manifestations (*p* ═ 0.03) and hepatitis (*p* ═ 0.001). Moderate negative correlation was proved between LV EF and proBNP (rr ═ −0.55; *p* < 0.001), as well as between LV EF and CRP (rr ═ −0.48; *p* < 0.001), LV EF and cTnI (rr ═ −0.46; *p* < 0.001), and LV EF and leukocyte count (rr ═ −0.313; *p* ═ 0.005) (Figure 4). A Z-score of the LV end-diastolic diameter (LF EDD) correlated positive with CRP (rr ═ 0.318; *p* < 0.001) and proBNP (rr 0.268; *p* ═ 0.02). A mild positive correlation was proved between CRP and proBNP and the Z-score of LV end-systolic diameter (LV ESD), a diameter of the interventricular septum and posterior wall (Table 2). The correlation between LV EF and cTnI was not proven. cTnI correlated positive with LV ESD Z-score (rr ═ −0.35, *p* ═ 0.008). LV systolic function was normalized in all patients during the hospital stay.

**Table 1:** Laboratory analysis on admission

**Laboratory analysis**	**Median value and IQR**
CRP (mg/L)	137 (97.0–247.0)
Leukocyte count (*10^9^)	10.53 (7.3–14.8)
Absolute number of lymphocytes (*10^9^)	900 (550–1550)
Platelet count (*10^9^)	145 (97–247)
Ferritin (ng/mL)	399 (182.7–753.7)
Sodium (mmol/L)	133 (131–135)
Albumin (g/L)	33 (31–37)
Phosphate (mmol/L)	1 (0.85–1.17)
LDH (IU/L)	549 (483–658)
SGOT (IU/L)	33 (22–56)
SGPT (IU/L)	32 (13.5–66.2)
Fibrinogen (g/L)	5.5 (3.75–6.6)
D-dimer (ng/mL)	952 (556–1950)
proBNP (pg/mL)	3303 (1183–5000)

CRP: C-reactive protein; LDH: Lactate dehydrogenase; SGOT: Serum glutamic-oxaloacetic transaminase; SGPT: Serum glutamic-pyruvic transaminase; proBNP: pro-B-type natriuretic peptide; IQR: Interquartile range.

A mild pericardial effusion with the spontaneous resolution was detected in 27.1% of children, while one female adolescent had severe pericardial effusion with threatening cardiac tamponade.

The shock was present in 21% of children on admission, while 4.9% developed shock during the hospital stay. Patients with shock were significantly older than those without shock (12.3 [SD 3.6] vs 8.4 [4.6] years; *p* < 0.001). All patients with shock except one had myocardial dysfunction (*p* < 0.001). Additionally, hepatitis was frequently observed in patients with shock (*p* ═ 0.054). Patients who presented with shock on admission had higher CRP (243, IQR 139.7–276.7 vs 122, IQR 87.2–195.5 mg/L; *p* ═ 0.005) and proBNP (5000, IQR 4330–15424 vs 1813, IQR 628.6–4635.0 pg/mL, *p* < 0.001), as well as lower sodium level than hemodynamically stable patients (130.3 [SD 3.8] vs 133.5 [3.1] mmol/L; *p* ═ 0.003).

CA dilatation was observed in 6.2% of patients; left CA (LCA) in 3 patients (Z-score +2.95 ± 0.3), right CA (RCA) in one patient (Z-score +2), and in LCA and RCA in one patient (LCA and RCA Z-score 2.6). Transient CA dilatations were observed only in patients with Kawasaki-like disease clinical presentation (5/55 patients with Kawasaki-like disease presentation developed transient CA dilatations).

On the standard ECG, 55.3% of children had negative T waves in inferior (85.7%) or/and precordial leads (28.5%). On average, negative T waves were observed on the second hospital day (median 2, IQR 1–3), but 57% of patients had negative T waves on admission. Transient QTc prolongation was registered in 43% of patients on average on day 7 (IQR 5–9). Sinus bradycardia and coronary rhythm were registered in 42.1% of patients, while monomorphic premature ventricular beats were observed in 2.4%.

A three-year-old boy with normal cardiac function was found to have an LV pedunculated thrombus (14×10 mm) in the apex. This patient had increased activity of factor VIII (280.5%, reference range 50%–150%) and factor XII (209.8%, reference range 50%–150%).

Patients with CV involvement (32/53) were more frequently hospitalized in PICU than patients without CV involvement (1/29; *p* < 0.001). Additionally, children with CV manifestations had longer hospital stays than patients without CV manifestations (11.5, IQR 9–14 vs 8, IQR 6.5–11 days; *p* < 0.001).

### Treatment

Inotropes were used in 25/81 patients: dopamine in 23, milrinone in 7, and dobutamine in 5 patients. Only one patient needed mechanical respiratory support.

**Figure 5.**
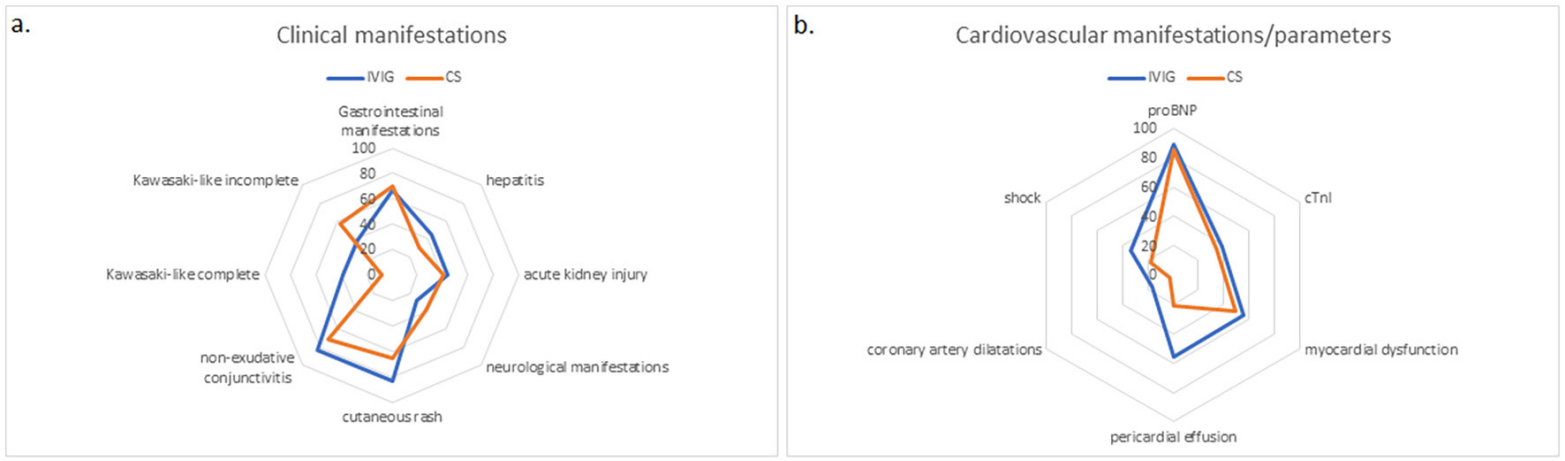
**Patients’ clinical and cardiovascular manifestations and parameters depended on initially used immunomodulatory drugs.** Radar graphs showing percentages of clinical (A) and cardiovascular manifestations (B). IVIG: Intravenous immunoglobulins; CS: Corticosteroids; cTnI: Cardiac troponin I; proBNP: pro-B-type natriuretic peptide.

In our study group, 63/81 (77.8%) patients were initially treated with CS, out of which 58 patients (92%) received IVMP. No difference in laboratory analysis and clinical presentation was observed between different treatment groups, whereas pericardial effusion was more frequent in patients treated with IVIG (*p* ═ 0.007). All patients with CA dilatations were treated with IVIG. Treatment failure was observed in 19 patients (23.4%). No difference between initial laboratory analysis was observed between children with and without treatment failure (*p* > 0.05) (Figure 5). Children with pericardial effusion had treatment failure more frequently (10/19; *p* ═ 0.08). Treatment failure was registered in 12/29 patients with neurological symptoms (*p* ═ 0.012). Only patients treated with IVIG developed shock syndrome during hospital stay (*p* ═ 0.02). Treatment failure was observed in 11/19 children initially treated with IVIG. Patients initially treated with IVIG had 10-fold higher chances for treatment failure than those treated with CS (OR 10.6, 95% CI 3.2–35.35; *p* < 0.001). Multinominal logistic regression analysis showed that IVIG treatment was an independent risk factor for treatment failure (OR 23.9, 95% CI 3.1–182.7; *p* < 0.001).

**Table 2:** Correlation between echocardiographic parameters and laboratory analyses

		**CRP**	**proBNP**	**cTnI**
LV EDD	Correlation coefficient	0.318	0.268	0.04
Z-score	*p* value	**0.004**	**0.02**	0.76
LV ESD	Correlation coefficient	0.362	0.394	0.355
Z-score	*p* value	**0.002**	**0.001**	**0.002**
LV IVS	Correlation coefficient	0.347	0.05	0.055
Z-score	*p* value	**0.005**	0.7	0.7
LV PW	Correlation coefficient	0.443	0.132	−0.212
Z-score	*p* value	**<0.001**	0.31	0.14

Bold values indicate statistical significance. CRP: C-reactive protein; proBNP: pro-B-type natriuretic peptide; cTnI: Cardiac troponin I; LV: Left ventricle; EDD: End-diastolic diameter; ESD: End-systolic diameter; IVS: Interventricular septum; PW: Posterior wall.

Patients with CV manifestations treated with IVIG had treatment failure more frequently (9/14) than patients with CV manifestations initially treated with CS (5/38; *p* ═ 0.001). On the other hand, in the group of patients without CV manifestations who were initially treated with CS, a trend toward a lower incidence of treatment failure was observed that approached statistical significance.

## Discussion

The pathogenesis of MIS-C is still insufficiently tested, but there are some hypothesis about this viral-associated autoimmune response: (1) molecular mimicry; (2) viral inducted epitope spreading; (3) liberation of self-antigens; (4) lower neutralization capacity (elevated antispike IgG and low antispike IgM levels); and (5) superantigens [1]. Since the immunopathogenesis of MIS-C is the virus-induced hyperimmune response, in patients, CD8+ T-cells are stimulated to kill virus-infected cells. On the other hand, in KD, the fundamental pathogenesis is the formation of antigen–antibody complexes [11].

The GI manifestations are the main manifestations in children who meet the criteria for MIS-C (almost 90% of patients with MIS-C), because the GI tract is a potential target of the viral immune-mediated inflammatory response. These children were more likely to have severe clinical courses, were more likely to require ICU admission, and had higher mortality rates, similar to our study [12]. On the other hand, feco-oral transmission and the essential binding receptors for SARS-CoV-2, ACE-2, and the protease-like transmembrane serine protease 2, in the human GI tract explain the self-limiting GI symptoms in COVID-19 in children, whereas 9.5% might have severe clinical course [24]. Although, some patients with long COVID or post-acute sequelae of COVID-19 might develop GI manifestations, in children with long COVID are frequently reported exercise intolerance, palpitations, headache, rhinitis, gustatory impairment, and appetite loss [13, 14]. Since our patients met the criteria for MIS-C and had favorable responses to applied immunomodulatory therapy, the GI symptoms are most likely due to a virus-triggered autoimmune response.

The CV manifestations were most frequently described in patients with MIS-C [4–7]. We found that patients with CV manifestation had significantly higher proinflammatory serum markers than patients without CV manifestation. Consequently, CV involvement has been seen in patients with the most complicated MIS-C form. We found a negative association between the degree of elevation of inflammatory markers and LV EF. Cytokine storm and endotoxemia may cause a decrease in LV EF, but some chemokines (CXCL9 and CXCL10) suggest a direct negative inotropic effect [2, 3]. Acute LV systolic dysfunction was found in more than half of MIS-C patients [4–7, 15, 16], similar to our study. In most cases, the EF was 30%–50%. On the other hand, lower myocardial strain values were more common in some patients than the LV EF reduction. Additionally, lower myocardial strain values could be seen in patients with a normal LV EF [5]. Myocardial dysfunction appears to be multifactorial. Literature shows that patients with MIS-C had myocardial stunning rather than cardiomyocyte necrosis [16]. Because of that, cTnI was less frequently detected in the blood samples than proBNP. Additionally, more than 70% of patients had complete recovery of LV function a median of two days after admission [4, 6, 10]. In children with myocardial dysfunction, treatment with CS was associated with more rapid normalization of fever, laboratory analysis, LV EF, and shorter ICU stay compared with IVIG treatment [10].

A mild to moderate pericardial effusion on echocardiogram was described in 13%–28% of MIS-C patients, but only sporadic cases presented with severe effusion [5, 17]. In our study group, 27% of patients had mild pericardial effusion. One of our patients had pericardial tamponade requiring emergency pericardiocentesis. Laboratory analysis indicated transudative pericardial effusion. One case similar to ours was described in [17].

The incidence of cardiogenic shock is much higher in MIS-C than in KD (40%–80% vs 2%–7%) [5]. In MIS-C patients, cardiogenic and vasoplegic shock were described [6]. In our study, all patients except one had moderate LV EF reduction. Additionally, patients who presented with shock had higher CRP and lower sodium levels than hemodynamically stable patients. The elevated inflammatory mediators increase vascular permeability, leading to fluid leakage into the extravascular compartment and decreasing sodium levels. Severe hyponatremia in KD is associated with more intense and protracted acute disease [9].

The etiopathogenesis of CA dilation in MIS-C has not been precisely explained. It may be linked to vasodilation due to fever, circulating inflammatory factors, and inflammation-induced hyperemia, or it might be the consequence of the vascular wall inflammation and disturbance of the arterial integrity, as seen in KD [1–3]. CA aneurysm prevalence in the MIS-C setting is 0%–48%, and mild dilation was most frequently described [4–8]. In our study, only 6.2% of patients had reversible mild CA dilatation, and all of these patients had Kawasaki-like disease clinical presentation. Giant CA aneurysms were described sporadically [18].

Arrhythmias and conduction abnormalities were frequently described in the MIS-C patients, with a wide dispersion of ECG changes, between 4% and 67% patients [5, 6, 19–21]. Abnormalities observed include repolarization changes and, in numerous cases, transitional QTc prolongations. The conduction abnormalities in MIS-C could be due to diffuse myocardial inflammation and edema in the transmission system. In addition, the ECG changes could be the result of inflammatory channelopathies [1, 4, 19, 20]. Although PR prolongation was the most common conduction delay in many studies, none of our patients had AV block.

Although hematological abnormalities are frequently described in MIS-C, thrombosis developed in a small number of patients [21]. Only one patient with reduced LV EF is described as having left ventricle thrombosis, while three patients with COVID-19 had normal LV EF, but all patients had central venous catheters [21–23]. Our patient with LV thrombosis had normal LV EF without contributing risk factors. In this patient, elevated activity of factor VII and factor XII was measured, which was previously described in hyperinflammatory states [21, 22].

MIS-C treatment includes supportive care and immunomodulatory drugs. Supportive care with balanced fluid resuscitation, mechanical respiratory support, inotropic drugs, and mechanical circulatory support in the most severe cases is fundamental in the acute phase of disease. Lo Vecchio et al. showed that 30% of patients required inotropic drug support, the same as in our study [12]. Most of our patients were treated with dopamine, while mechanical respiratory support was used in one patient. Numerous studies have shown that the addition of CS to IVIG leads to faster recovery [9, 24]. In our study, IVIG treatment was an independent risk factor for treatment failure in all groups of patients. We also proved that IVIG-treated patients with CV manifestations had more frequently treatment failure than patients treated with CS. Consequently, that might explain why McArdle et al. [25] concluded that the primary treatment (IVIG or CS alone or IVIG plus CS) did not influence the recovery from MIS-C. Namely, McArdle et al. analyzed all patients with MIS-C, while Belhajder et al. included only patients with MIS-C who had acute myocardial dysfunction [9, 21]. Additionally, our IVIG-treated patients developed shock more frequently, while none patient treated with CS developed shock. Vasoconstrictive effects of CS could explain this due to endothelial inhibition, which is an essential CS anti-inflammatory effect [26].

Due to the differences in the immunopathogenesis of MIS-C and KD, different responses to the applied therapy could be expected. Namely, IVIG in KD bind to inflammatory cells’ Fc receptors, preventing cell activation and ameliorating the inflammatory response triggered by antigen-antibody complexes [11]. On the other hand, the activation of T-cells triggered by SARS-CoV-2 leads to a cytokine and chemokine storm, that CS could better control, because CS change the traffic patterns of those cells. After a bolus of CS, there is an increase in blood neutrophils and a decrease in lymphocytes, monocytes, eosinophils, and basophils. Lymphocyte count is decreased primarily due to sequestration in the reticuloendothelial system. Additionally, the function of the inflammatory cells is impaired by CS [26].

### Limitation of the study

The major limitation of the study is its retrospective design. Additionally, the other most significant limitation of the study is the small number of patients, which makes it difficult to derive definitive recommendations for MIS-C treatment. It is necessary to conduct randomized multicenter studies to establish treatment protocols with a significant level of recommendations.

## Conclusion

MIS-C temporally associated with COVID-19 is a rare but severe hyperimmune reaction with widespread CV abnormalities. CV involvement was observed in 64% of patients, with acute myocardial dysfunction being the most common. Our study showed that patients treated with CS had a lower risk of treatment failure than patients treated with IVIG, so we strongly recommend the use of CS in the treatment of MIS-C. CS should be the first choice, especially in patients with CV manifestations in MIS-C temporally associated with COVID-19.
